# Spleno-adrenal fusion mimicking an adrenal metastasis of a renal cell carcinoma: A case report and embryological background

**DOI:** 10.1515/med-2021-0201

**Published:** 2020-12-23

**Authors:** Zbyněk Tüdös, Paulína Szász, Lucia Veverková, František Hruška, Igor Hartmann, Jozef Škarda, Rohit Philip Thomas

**Affiliations:** Department of Radiology, University Hospital and Faculty of Medicine and Dentistry, Palacky University, I. P. Pavlova 6, 77900 Olomouc, Czech Republic; Department of Urology, University Hospital and Faculty of Medicine and Dentistry, Palacky University, I. P. Pavlova 6, 77900 Olomouc, Czech Republic; Department of Clinical and Molecular Pathology, University Hospital and Faculty of Medicine and Dentistry, Palacky University, Hněvotínská 3, 775 15 Olomouc, Czech Republic; Department of Diagnostic and Interventional Radiology, University Hospital Marburg, Philipps University, Baldingerstrasse, 35043 Marburg, Germany

**Keywords:** accessory spleen, adrenal gland neoplasms, spleno-adrenal fusion, spleno-gonadal fusion, spleno-renal fusion, splenic heterotopy, splenosis

## Abstract

Foci of splenic tissue separated from the spleen can occur as a congenital anomaly. Isolated nodules of splenic tissue are called accessory spleens or spleniculli. However, nodules of splenic tissue can merge with other organs during embryonic development, in which case we speak of spleno-visceral fusions: most often, they merge with the tail of the pancreas (thus forming spleno-pancreatic fusion or an intrapancreatic accessory spleen), with the reproductive gland (i.e., spleno-gonadal fusion), or with the kidney (i.e., spleno-renal fusion). Our case report describes the fusion of heterotopic splenic tissue with the right adrenal gland, which was misinterpreted as a metastasis of a renal cell carcinoma. To the best of our knowledge, this is the first reported case of spleno-adrenal fusion. Spleno-visceral fusions usually represent asymptomatic conditions; their main clinical significance lies in the confusion they cause and its misinterpretation as tumors of other organs. We believe that the cause of retroperitoneal spleno-visceral fusions is the anomalous migration of splenic cells along the dorsal mesentery to the urogenital ridge, together with primitive germ cells, at the end of the fifth week and during the sixth week of embryonic age. This theory explains the possible origin of spleno-visceral fusions, their different frequency of occurrence, and the predominance of findings on the left side.

## 1 Introduction

Congenital heterotopia of splenic tissue can occur in the form of isolated accessory spleens, which are relatively common, or in the form of spleno-visceral fusions, i.e., the association of splenic tissue with another abdominal organ. These are asymptomatic conditions; their main clinical significance lies in the confusion they cause and its misinterpretation as tumors of other organs. So far, cases or even smaller groups of patients with spleno-pancreatic, spleno-gonadal, and spleno-renal fusion have been published. Our article presents a case report of fusion of heterotopic splenic tissue and the right adrenal gland. The finding was mistaken for an adrenal metastasis of a renal carcinoma of the right kidney; the correct diagnosis was made by histology after an adrenalectomy. In the discussion, we present a possible embryological basis for this anomaly, but also for other spleno-visceral fusions in the retroperitoneum.

## 2 Case report

**Figure 1:**
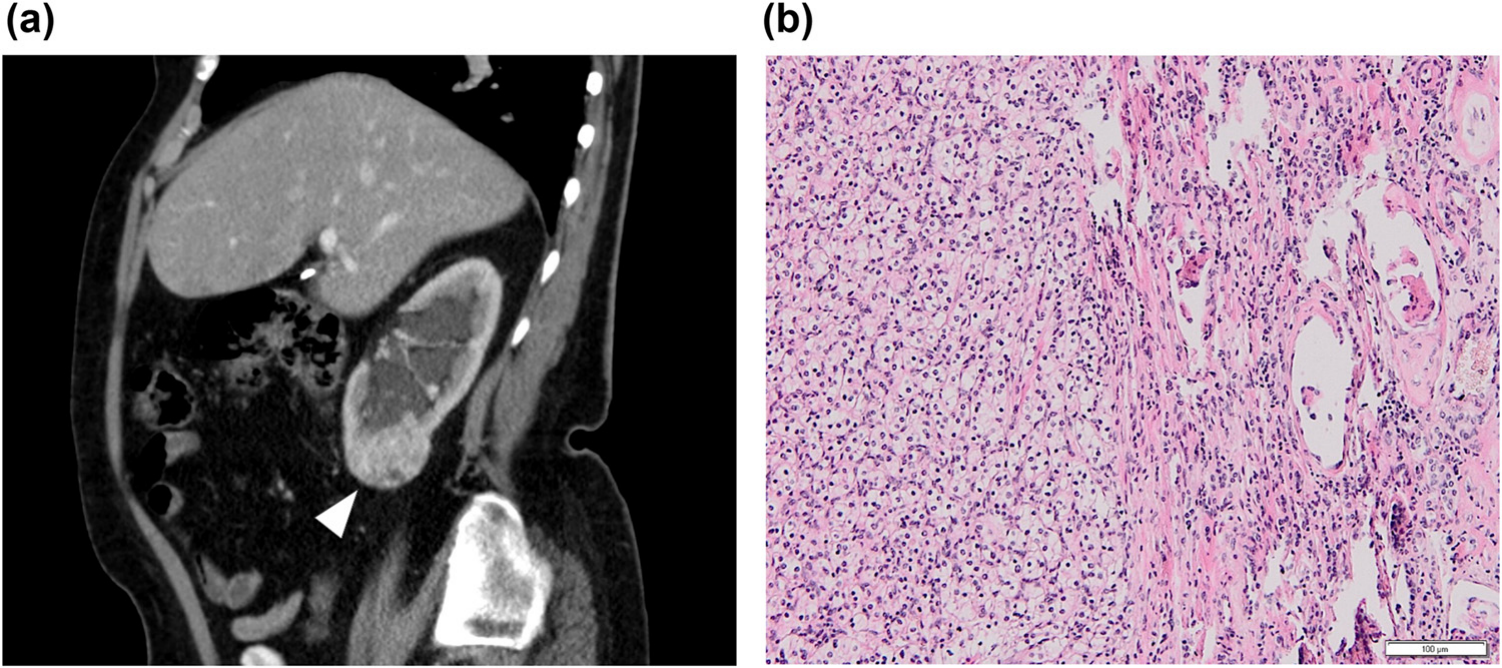
(a) Abdominal CT scan revealing a mass on the lower pole of the right kidney (arrowhead) in the sagittal plane. (b) Histology confirmed the renal tumor to be a conventional renal cell carcinoma grade I–II (hematoxylin & eosin, 100×).

**Figure 2:**
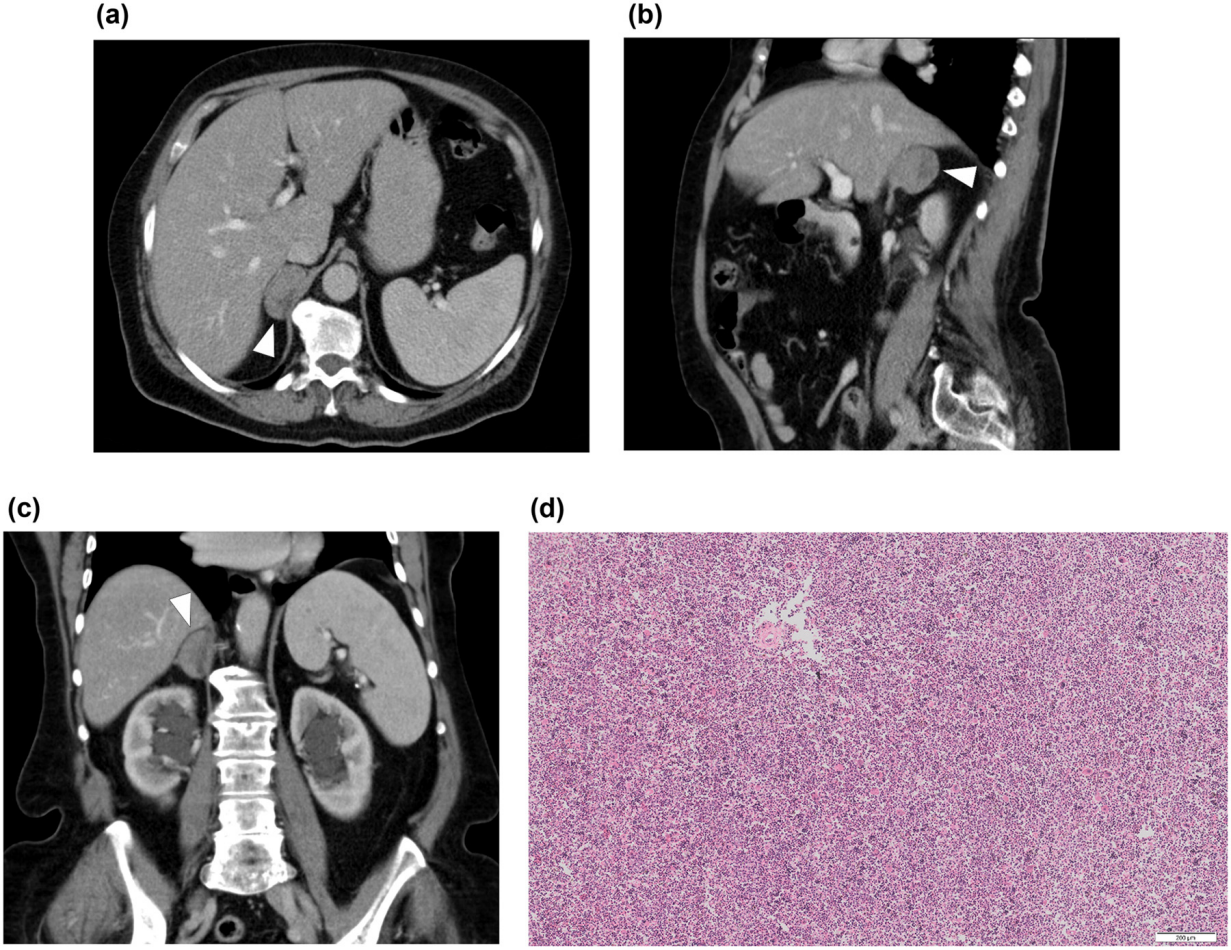
Abdominal CT scan revealing the mass of the right adrenal gland (arrowheads) in (a) the axial plane, (b) the sagittal plane, and (c) the coronal plane. Note the orthotopic intact spleen. There are clips after a cholecystectomy in the hepatic hilum and parapelvic cysts of the right kidney as additional findings. (d) Histology proved heterotopic splenic tissue connected to the right adrenal gland. The sample included a large proportion of red pulp with the presence of extramedullary hematopoiesis; damaged white pulp with a central artery is also present (hematoxylin & eosin, 100×).

A 79-year-old female patient with dyspnea underwent computed tomography (CT) angiography to rule out pulmonary embolism in October 2017. There was only arterial hypertension, bilateral hip arthroplasty, and laparoscopically assisted cholecystectomy in the patient’s history. A mass on the right adrenal gland was incidentally detected on the CT scan. With this finding, the patient was referred to a urological department in a tertiary medical center. A lesion of the right adrenal gland was confirmed on the subsequent abdominal CT, and in addition, another incidental lesion was found on the right kidney. A tumor on the lower pole of the right kidney measured 45 × 38 × 34 mm, with significant heterogeneous enhancement; it was interpreted as a suspected renal cell carcinoma (Figure 1a). The mass on the right adrenal gland had dimensions of 41 × 38 × 24 mm (Figure 2a–c); its attenuation was 41 Hounsfield units (HU) in the unenhanced phase, 71 HU in the venous phase, and 64 HU in the late phase; the absolute percentage rate was 23.3% and the relative percentage rate was 9.9%. Neither the unenhanced attenuation nor the values of the wash-out rate indicated a typical adenoma; therefore, the lesion had to be classified as indeterminate. Endocrinological examination did not reveal increased hormonal activity. With regard to the finding of a tumor of the right kidney, metastasis seemed to be the most likely. Unfortunately, about 20% of patients with a malignant renal tumor are diagnosed in the metastatic stage [1]. A suspected malignant renal tumor should be removed with clear surgical margins [2,3]. In the case of cT1 renal neoplasm, partial nephrectomy or enucleation is recommended [4,5] in order to preserve renal function [3] for eventual adjuvant therapy. Therefore, a combined surgical procedure was indicated to remove both the renal and adrenal mass in December 2017. First, a robot-assisted partial nephrectomy was performed on the right kidney using a 5-port transperitoneal approach; the warm ischemia time was 19 min. A right-sided robot-assisted adrenalectomy followed directly. The patient was lying in the left lateral decubitus position, the overall operating time was 95 min and blood loss was minimal. The postoperative course was uneventful and the patient was discharged on the fifth postoperative day.

Histological examination of the right kidney tissue confirmed a clear cell renal carcinoma pT1b, N0, M0, grade I–II (Figure 1b). The removed adrenal gland was without any metastatic involvement, but the cortical layer of its medial limb was fused with a nodule surprisingly formed by the splenic parenchyma with a large proportion of red pulp with the presence of extramedullary hematopoiesis (Figure 2d). In the case of hematopoietic elements included inside an adrenal tumor, it is necessary to consider the differential diagnostic possibility of an adrenal myelolipoma, which is a benign tumor-like lesion composed of mature adipose tissue admixed with hematopoietic elements in various proportions [6]. In our case, the sample contained splenic white pulp; on the other hand, it did not contain a fat component, and therefore, it was possible to rule out a myelolipoma.

The patient underwent follow-up abdominal CT in July 2018; the finding was without recurrence of the tumor. At the last follow-up clinical check in March 2020, the patient felt in good health condition.

Regarding the management of such a case, we believe that spleno-adrenal fusion is an extremely rare anomaly and could not be predicted a priori. In similar conditions that are more common in clinical practice, such as splenosis or accessory spleens mimicking a neoplastic lesion, the suspicion may be confirmed by 99m Technetium heat-damaged red blood cell scintigraphy combined with single-photon emission CT.

The patient provided an informed consent to all the diagnostic and therapeutic procedures. Further, the patient provided a general informed consent to the use of the results of the examination methods for the purposes of research and publication, which is included as a part of the informed consent for hospitalization in our department.

## 3 Discussion

### 3.1 Splenic heterotopy and the development of the spleen

Foci of splenic tissue separated from the main body of the spleen can occur for several reasons. In general, they are considered asymptomatic and only very rarely is their presence accompanied by complications, such as torsion of a wandering accessory spleen or bleeding caused by spontaneous rupture [7]. The main clinical problem is their differential diagnostic confusion with tumor lesions. Although spleen tissue foci may occur in different areas of the abdomen and chest, in this discussion we will focus on their occurrence in the region of the retroperitoneum and retroperitoneal organs.

Splenosis represents one of the possible causes of foci of splenic tissue occurring in different regions of the body. It is not a congenital condition, but an autotransplantation of the splenic parenchyma at the time of trauma or a splenectomy [8]. If a focus of splenosis occurs in the suprarenal area, it can be confused with an adrenal tumor; such cases have already been described in the literature [8,9,10]. As already mentioned, splenosis should be considered in a patient with a history of splenic trauma or splenectomy. Heterotopy of the splenic tissue may be congenital.

**Figure 3:**
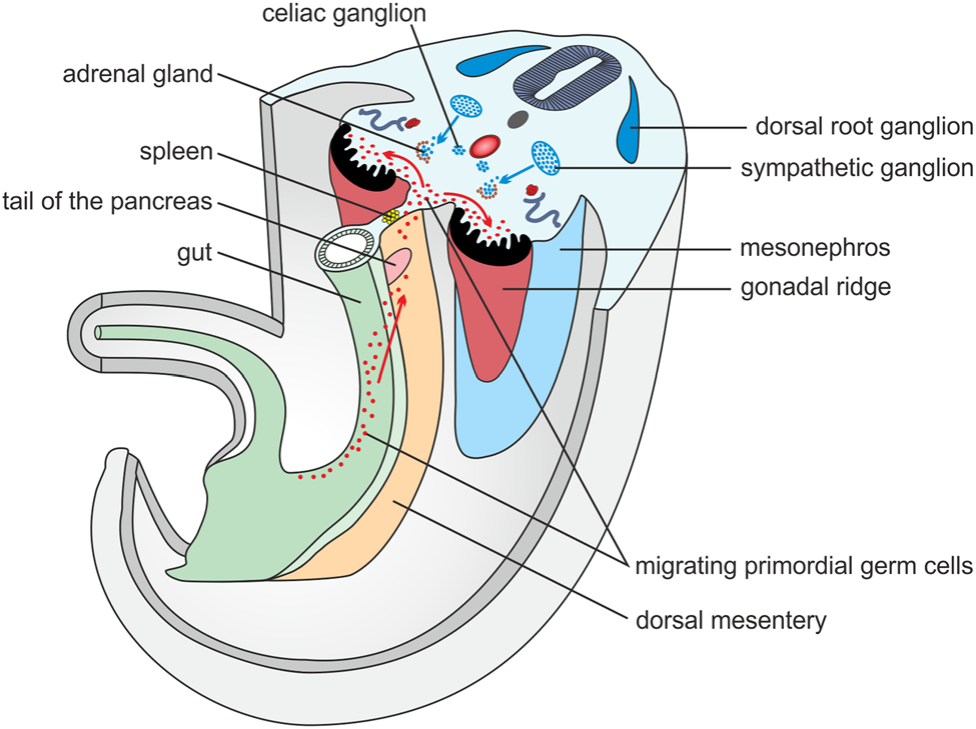
A scheme showing a transsection of an embryo during the fifth week of embryonic age. Note the primordial germ cells migrating along the dorsal mesentery into the retroperitoneum in close proximity to the developing spleen.

The spleen is derived from a mass of mesenchymal cells located between the layers of the dorsal mesogastrium and begins to develop during the fifth week [11]. The proliferating cells invade the underlying angiogenetic mesenchyme, which becomes condensed and vascularized. The process occurs simultaneously in several adjoining areas, which soon fuse to form a lobulated fetal spleen (Figure 3) [12]; the lobules normally disappear before birth [11]. Accessory spleens may be formed during embryonic development as heterotopic splenic tissue [12]; the occurrence of such a variety is reported in 4–15% of the population [12]. The most common localization is in the hilum of the main spleen, the great omentum, the gastrosplenic or spleno-renal ligament, and the pelvis [12]. It should also be emphasized that in humans ectopic splenic tissue also has a different architecture compared to a normal spleen, with plenty of red pulp and quite a small area of white pulp [13,14]. Accessory spleens may represent a differential diagnostic challenge, and several cases of an accessory spleen being mistaken for an adrenal tumor have been published [15,16,17]. The very rare accessory spleens mimicking a retroperitoneal tumor on the right side represent an even more challenging situation [18,19,20]. If a developmentally separate heterotopic splenic nodule is directly connected to another organ, the term “spleno-visceral fusion” should be used instead of “accessory spleen;” however, there is inconsistency in the literature and the term “accessory spleen” is also used in this context [21].

### 3.2 Spleno-pancreatic fusion and the development of the pancreas

Compared to other types of spleno-visceral fusions, fusion with the pancreas is probably the most common. Unver Dogan et al. report the occurrence of heterotopic splenic tissue within the tail of the pancreas in 0.7% of autopsies [12]. Case reports of splenic tissue being mistaken for a tumor of the pancreatic tail have also been published [22,23] and criteria to distinguish the two using magnetic resonance imaging have been proposed [21].

The embryological explanation is relatively straightforward, as the spleen and tail of the pancreas have their embryological origin in the dorsal mesogastrium during the fifth week and lie in relatively close proximity to one another (Figure 3) [11]. This fact may also explain the relatively high frequency of this type of fusion.

### 3.3 Spleno-gonadal fusion and the development of gonads

The connection of the spleen and reproductive glands is the second most common form of fusion; so far about 175 cases have been described [24]. Spleno-gonadal fusion can be classified into continuous or discontinuous types on the basis of the presence or absence of a continuous band of splenic tissue or a fibrous cord connecting the spleen and the gonad [25]. Out of 84 spleno-gonadal fusions, the majority were described in boys, and the gender difference is explained by the better availability of testes for clinical examination [25,26]. Only isolated cases are described on the right side [25,27]. The anomaly occurs in two forms, allowing division of the cases into two major subgroups: (1) continuous spleno-gonadal fusion, in which a continuous cord-like structure connects the spleen and the gonadal-mesonephric structures; (2) discontinuous spleno-gonadal fusion, in which the fused spleno-gonadal-mesonephric structures have lost continuity with the main spleen [28]. The continuous type is often associated with other congenital anomalies, especially of the limbs and lower jaw [25,28]. On the contrary, a single case classified as being the discontinuous type associated with other anomalies was described [25]. Thus, there is a theory according to which continuous spleno-gonadal fusion, with its associated high incidence of limb and jaw defects, is probably caused by a teratogenic insult between the fifth and eighth weeks of embryonic age, whereas discontinuous spleno-gonadal fusion represents a rare variant of an accessory spleen [29]. Thus, the theories of the embryological basis of spleno-visceral fusions below probably relate only to the discontinuous fusion.

The initial stages of gonadal development occur during the fifth week, when a thickened area of the mesothelium develops on the posterior abdominal wall on the medial side of the mesonephros. The proliferating epithelium and underlying mesenchyme create a bulge called the genital ridge (Figure 3) [30]. Primordial germ cells are large, spherical sex cells and originate from the endodermal cells of the umbilical vesicle near the origin of the allantois. During the fourth and fifth weeks, primordial germ cells migrate by amoeboid movement along the dorsal mesentery of the hindgut to the gonadal ridges, where they arrive during the sixth week (Figure 3) [30]. Approximately in the eighth week, the gonadal glands start to descend into the pelvis [30].

### 3.4 Spleno-renal fusion and the development of the kidney

Congenital connections of heterotopic splenic tissue and the kidney are much rarer in the literature than are those of the reproductive organs; so far about 13 cases have been published, including four in the right kidney [31,32,33,34,35,36].

Three slightly overlapping kidney systems are formed in the urogenital ridge during intrauterine life. The first of these systems, called the pronephros, is rudimentary and nonfunctional. It is presented during the fourth week [37]. The second system, called the mesonephros, starts to develop at the end of the fourth week and is temporarily functional until approximately the tenth week (Figure 3). The third kidney system, called the metanephros, forms the permanent kidney and develops between the fifth and the tenth week [37]. From the fifth to the ninth week, the permanent kidneys change their relative position in the caudal direction because of disproportional growth of the embryo’s body [37]. According to Rosenthal et al., there are several predisposing conditions for the formation of spleno-renal fusion. It must occur between the sixth and the ninth week of development, when the kidney reaches its final position [31]. Alternatively, the holonephric theory of the blurring of the distinction among the pronephros, mesonephros, and metanephros in renal development or the migration of splenic cells via blood supply could explain spleno-renal fusion at a later stage [31].

### 3.5 Spleno-adrenal fusion and the development of the adrenal gland

Congenital connections of heterotopic splenic tissue and the adrenal gland have been theoretically assumed, but we were unable to find any documented case of spleno-adrenal fusion. There are several case reports of accessory spleens in the right suprarenal space misinterpreted as retroperitoneal neoplasms, but there was no direct connection to the right adrenal gland [18,19,20]. As already mentioned, an adrenal myelolipoma should be considered and ruled out if an adrenal tissue sample contains hematopoietic elements. The adrenal gland develops from two components: a mesodermal portion, which forms the cortex, and an ectodermal portion, which forms the medulla. During the fifth week of development, mesothelial cells between the root of the mesentery and the developing gonad at the level of the celiac plexus begin to proliferate and penetrate the underlying mesenchyme to create the fetal cortex (Figure 3) [38,39]. The second wave of mesothelial cells creates the definitive cortex [39]. While the fetal cortex is being formed, neural crest cells originating in the adjacent sympathetic ganglion invade its medial aspect, where they are arranged in cords and clusters (Figure 3). These cells give rise to the medulla of the adrenal gland [39].

### 3.6 The hypotheses explaining spleno-visceral fusion

Several theories have been proposed that attempt to explain the embryological background of retroperitonal spleno-visceral fusions. To the best of our knowledge, Sneath came up with the first hypothesis in 1913 [40]. He pointed out that the spleen, as it is developed in the dorsal mesogastrium, lies in apposition to the urogenital ridge lying upon the posterior wall of the abdomen during the sixth week. He assumed that slight inflammation of the peritoneum may cause an adhesion to be formed between the spleen and the genital gland [40]. It should be emphasized that Sneath postulated his hypothesis to clarify the case of left-sided continuous spleno-testicular fusion. The adhesion hypothesis of the origin of spleno-gonadal fusion was later accepted by other authors, with special emphasis on the fragility of the spleen, e.g., by Talmann in 1926 [41]. The fragility of the spleen was reported to result in the loss of its tissue along the path of development during the mechanical injuries caused by the adhesions. However, this theory of simple adhesion has its weak points. Specifically, it does not explain the findings of splenic tissue nesting deep inside the parenchyma of other organs. And further, a right-sided finding would remain unexplained. In 1953, Hochstetter published his theory, which sought to explain the occurrence of heterotopic splenic tissue inside the left ovary of a left-sided thoracopagus twin specimen [42]. He assumed the migration of splenic cells along the caudal limiting fold, which is the medial and most caudal portion of the pleural-peritoneal membrane (Septum pleuroperitoneale mediale), which would provide a direct route from the dorsal mesogastrium to the retroperitoneum [42]. However, as Rosenthal et al. have noted, Hochstetter’s hypothetical way of migration does not explain the rare findings of right-sided fusions since the caudal limiting fold provides access only to the left retroperitoneum, and in addition, the hypothesis could explain the finding on the ovaries, but it is difficult to apply to other retroperitoneal organs [31]. Alternatively, Rosenthal suggests the migration of splenic cells along the dorsal mesentery [31]. According to Rosenthal et al., the larger proportion of left-sided findings could be explained by the migration of splenic cells after the beginning of the rotation of the digestive tract, i.e., at the end of the fifth and during the sixth week [31]. Later, Gouw et al. correctly pointed out in the discussion of their paper the need for the migration of primordial germ cells through the dorsal mesentery to reach the urogenital ridge [25].

In the context of the above facts, we offer an interpretation of our own finding. In our case, with histologically proven splenic tissue within the right adrenal gland, we primarily ruled out the possibility of splenosis, as there was no splenic trauma or splenectomy in the patient’s history (note the intact spleen in Figure 2a and c). We therefore considered our finding to be a congenital heterotopy. Accessory spleens in the right suprarenal space misinterpreted and removed as retroperitoneal neoplasms have been published, but these cases do not mention a direct relationship of splenic tissue to the adrenal gland [18,19,20]. We have not found any previous mention of spleno-adrenal fusion in the literature. We therefore believe that our patient is the first case of spleno-adrenal fusion to be documented using modern imaging methods. From published theories of how spleno-adrenal fusion in the right adrenal gland occurred, we can rule out the theories of Sneath and Hochstetter [40,42]. The only possible way is the migration of splenic cells along the dorsal mesogastrium after the beginning of the rotation of the digestive tract, as described by Rosenthal [31]. In general, we believe that the cause of retroperitoneal spleno-visceral fusions (with the exception of spleno-pancreatic fusions, which can be explained by the proximity of both organs in the dorsal mesentery) is splenic cell migration, together with primitive germ cells at the end of the fifth and during the sixth week (Figure 3). This theory possibly explains the different frequency of splenic fusion with different retroperitoneal organs, i.e., in most cases splenic cells arrive in the area of the genital ridge together with primitive germ cells (dozens to hundreds of described cases), a small number arrive in the area of the mesonephros (about 13 described cases), and only very rarely do they arrive in the adrenal area (three reported cases of an accessory spleen in the right retroperitoneum and our single case of spleno-adrenal fusion). The time of the migration of primitive germ cells also corresponds to the beginning of the rotation of the digestive tract and mesogastrium, which explains the mostly left-sided findings. The reason why splenic cells follow primitive germ cells still remains unclear.

### 3.7 Imaging diagnosis of splenic heterotopy

Spleno-renal and discontinuous spleno-gonadal fusion are usually mistaken for a potentially malignant kidney or testicular tumor, as splenic heterotopy in these organs is a very rare condition and the finding of imaging methods is nonspecific. Also in our case of spleno-adrenal fusion, the lesion was evaluated as an indeterminate adrenal mass and there was a high probability of metastatic adrenal involvement when a renal cell carcinoma was suspected. For spleno-pancreatic fusion or an intrapancreatic accessory spleen, which is relatively more common than other fusions, criteria have been proposed to distinguish the splenic tissue from tumors of the pancreatic tail [23,43]. In the case of splenosis, there is a diagnostic clue of splenic injury or splenectomy in a patient’s history. If splenic tissue heterotopy or splenosis is suspected, 99m Technetium heat-damaged red blood cell scintigraphy combined with single-photon emission CT is the method of choice to confirm the diagnosis, as it offers excellent specificity [44]. It is also well-established in distinguishing an intrapancreatic accessory spleen from pancreatic tumors [45,46,47].

## 4 Conclusion

To the best of our knowledge, this is the first reported case of spleno-adrenal fusion. We believe that the cause of retroperitoneal spleno-visceral fusions is the anomalous migration of spleen cells along the dorsal mesentery to the urogenital ridge, together with primitive germ cells, at the end of the fifth and during the sixth week of embryonic age. This theory explains the possible origin of spleno-visceral fusions, their different frequencies of occurrence, and the predominance of findings on the left side. We believe that spleno-adrenal fusion is an extremely rare anomaly and could not be predicted a priori. In similar conditions that are more common in clinical practice, such as splenosis or accessory spleens mimicking a neoplastic lesion, the suspicion may be confirmed by 99m Technetium heat-damaged red blood cell scintigraphy combined with single-photon emission CT.
